# Monkeypox: another test for PCR

**DOI:** 10.2807/1560-7917.ES.2022.27.32.2200497

**Published:** 2022-08-11

**Authors:** Jim F Huggett, David French, Denise M O’Sullivan, Jacob Moran-Gilad, Alimuddin Zumla

**Affiliations:** 1 National Measurement Laboratory, LGC, Teddington, United Kingdom; 2 School of Biosciences and Medicine, Faculty of Health and Medical Sciences, University of Surrey, Guildford, United Kingdom; 3 Department of Health Policy and Management, School of Public Health, Faculty of Health Sciences, Ben Gurion University of the Negev, Beer Sheva, Israel; 4 Department of Clinical Microbiology and Infectious Diseases, Hadassah Medical Center, Jerusalem, Israel; 5 Centre for Clinical Microbiology, Division of Infection and Immunity, University College London, United Kingdom; 6 National Institutes of Health and Research Biomedical Research Centre, University College London Hospitals NHS Foundation Trust, London, United Kingdom

**Keywords:** Monkeypox, molecular diagnosis, PCR, standardisation, test accuracy

## Abstract

Monkeypox was declared a public health emergency of international concern by the World Health Organization (WHO) on 23 July 2022. Between 1 January and 23 July 2022, 16,016 laboratory confirmed cases of monkeypox and five deaths were reported to WHO from 75 countries on all continents. Public health authorities are proactively identifying cases and tracing their contacts to contain its spread. As with COVID-19, PCR is the only method capable of being deployed at sufficient speed to provide timely feedback on any public health interventions. However, at this point, there is little information on how those PCR assays are being standardised between laboratories. A likely reason is that testing is still limited on a global scale and that detection, not quantification, of monkeypox virus DNA is the main clinical requirement. Yet we should not be complacent about PCR performance. As testing requirements increase rapidly and specimens become more diverse, it would be prudent to ensure PCR accuracy from the outset to support harmonisation and ease regulatory conformance. Lessons from COVID-19 should aid implementation with appropriate material, documentary and methodological standards offering dynamic mechanisms to ensure testing that most accurately guides public health decisions.

## Introduction

From 1 January through 22 July 2022, 16,016 laboratory confirmed cases of monkeypox and five deaths were reported to the World Health Organization (WHO) from 75 countries [1]. The WHO declared the current outbreak a public health emergency of international concern (PHEIC) on 23 July. Public health authorities are proactively identifying cases, and tracing contacts, to contain its spread, yet it may have been spreading undetected in Europe and other previously non-endemic areas for a while [2]. Like COVID-19, monkeypox diagnosis depends on PCR. As PCR assays are increasingly being deployed to aid management of this growing threat, it would be prudent to ensure the lessons learned from the early diagnostic response to COVID-19 are applied to monkeypox.

PCR is a rapidly deployable and versatile approach, but it must be applied with care if it is to accurately identify patients and aid contact tracing. The versatility of PCR can also be its weakness; notwithstanding the immense public health benefits of the rapid development and roll-out of PCR in the face of a new pandemic, there were certain challenges in the early response to COVID-19, such as contamination problems [3] and varying sensitivities reportedly differing by > 1,000-fold [4]. Such challenges are avoidable when responding to monkeypox as lessons from COVID-19 can guide us in the fast design, deployment and standardisation diagnostic PCR to be most effective.

## Approaching PCR assay design and basic setup

Unlike severe acute respiratory syndrome coronavirus 2 (SARS-CoV-2) in early 2020, monkeypox virus is not a new virus and several published assays are already available [5-11], some developed by diagnostics manufacturers. When choosing a PCR assay to detect monkeypox, in silico tools can be used to predict their performance and aid selection (Table); Supplementary Table S1 provides a detailed list of in silico-analysed targets collated by the authors; the underlying method and choice of targets are described in the Supplement. While in silico analysis increases the chances a selected assay will perform well, it cannot replace wet laboratory assessment. Monkeypox virus shares genetic similarities with other viruses in the *Poxviridae* family, however, as these other family members are generally rare their undesired detection is not likely to impact on test specificity. Members of the *Poxviridae* family have very large genomes with many potential PCR targets of varying specificities: assays can range from pan-*Orthopoxvirus* detection [8] to specific detection of monkeypox virus [9] (Table); see Supplementary Table S1 for a more detailed analysis of targets. The cause of chickenpox, varicella zoster virus, which may result in clinically suspected cases of monkeypox, is an unrelated herpesvirus that should not be detectable by an *Orthopoxvirus*-specific diagnostic PCR. The most likely adverse effect of a poorly designed and/or poorly optimised monkeypox PCR assay will be on the limit of detection (LOD), leading to false-negative results and reduced analytical sensitivity. 

**Table:** Examples^a^ of published PCR assays to detect monkeypox virus

Gene target	Targeted lineage	Oligonucleotide	Sequence^b^	T_m_ in °C ^c^	Reference
F3L	Monkeypox generic	F	CTCATTGATTTTTCGCGGGATA	63.6	[11]
		R	**G** ACGATACTCCTCCTCGTTGGT	67.0	
		P	6FAM-CATCAGAATCTGTAGGCCGT-MGBNFQ	60.5	
N3R	Monkeypox generic	F	AACAACCGTCCTACAATTAAACAACA	66.0	
		R	CGCTATCGAACCATTTTTGTAGTCT	65.4	
		P	FAM-TATAACGGCGA **A** GAATATACT-MGBNFQ	55.9	
E9L-NVAR	Eurasian orthopox (non-variola)	F	TCAACTGAAAAGGCCATCTATGA	64.2	[10]
		R	GAGTATAGAGCACTATTTCTAAATCCCA	64.1	
		P	TET-CCATGCAATATACGTACAAGATAGTAGCCAAC-QSY7	67.5	
B6R	Monkeypox generic	F	ATTGGTCATTATTTTTGTCACAGGAACA	66.3	
		R	AATGGCGTTGACAATTATGGGTG	65.9	
		P	MGB/DarkQuencher-**A** GAGATTAGAAATA-FAM	39.2	
ATI	Congo	F	GAGATTAGCAGACTCCAA	57.9	[6]
		R	GATTCAATTTCCAGTTTGTAC	57.9	
		P1	GCAGTCGTTCAACTGTATTTCAAGATCTGAGAT-Fluorescein	68.9	
		P2	LCRed640-CTAGATTGTAATCTCTGTAGCATTTCCACGGC-Phos	68.6	
ATI	West African	F	GAGATTAGCAGACTCCAA	57.9	
		R	TCTCTTTTCCATATCAGC	56.2	
		P1	GCAGTCGTTCAACTGTATTTCAAGATCTGAGAT-Fluorescein	68.9	
		P2	LCRed640-CTAGATTGTAATCTCTGTAGCATTTCCACGGC-Phos	68.6	
G2R_WA	Monkeypox West African-specific	F	CACACCGTCTCTTCCACAGA	65.4	[9]
		R	GATACAGGTTAATTTCCACATCG	61.4	
		P	FAM-AACCCGTCGTAACCAGCAATACATTT-BHQ1	67.8	
G2R_G	Monkeypox generic	F	GGAAA **A** TGTAAAGACAACGAATACAG	62.9	
		R	GCTATCACATAATCTG **G** AAGCGTA	64.2	
		P	FAM-AAGCCGTAATCTATGTTGTCTATCGTGTCC-BHQ1	68.2	
C3L	Monkeypox Congo basin-specific	F	TGTCTACCTGGATACAGAAAGCAA	65.4	
		R	GGCATCTCCGTTTAATACATTGAT	63.4	
		P	FAM-CCCATATATGCTAAATGTACCGGTACCGGA-BHQ1	69.1	
B7R	Monkeypox generic	F	ACGTGTTAAACAATGGGTGATG	63.3	[5]
		R	AACATTTCCATGAATCGTAGTCC	62.7	
		P	TAMRA-TGAATGAAT **G** CGATACTGTATGTGTGGG-BHQ2	67.8	
F3L	Monkeypox generic	F	CATCTATTATAGCATCAGCATCAGA	62.1	[7]
		R	GATACTCCTCCTCGTTGGTCTAC	64.3	
		P	JOE-TGTAGGCCGTGTATCAGCATCCATT-BHQ1	68.8	

F: forward; P: probe; R: reverse; T_m_: estimated melting temperature;

^a^ The methodological approach to the in silico analysis and choice of examples are described in the Supplement.

^b^ Sequence positions of mismatch compared with the NCBI Virus alignment and NC_003310.1 sequence are highlighted in bold and underlined.

^c^ Oligonucleotide T_m_ were determined with the Integrated DNA Technologies OligoAnalyzer tool using the defined oligonucleotide and Mg^2+^ concentrations. The Na^+^ and dNTP concentrations were 50 mM and 0.8 mM, respectively. Calculated T_m_ do not account for the identified positions of mismatch.

To mitigate against false-negative results associated with design or optimisation, laboratories should always evaluate published protocols by using control materials and/or clinical specimens with their own reagents and instruments; it is not appropriate to simply repeat the published protocols verbatim. Analytical performance evaluations, such as the LOD, should be performed with accurately quantified control materials that are ideally similar to the clinical specimen of choice (see description of reference materials below). Given the current state of the art, it can be expected that, once the pre-analytical factors are accounted for, the PCR amplification step of the procedure will be capable of detecting small amounts (copy numbers) of DNA per reaction. Well-designed and optimised assays could be expected to demonstrate near single-copy performance. 

False-positive results will primarily be caused by contamination. In viral diagnostic PCRs, there are two broad sources of potential contamination: specimens with high titres and synthetically derived template. Consequently, when specimens from positive and negative patients are processed together, cross-contamination from the former to the latter is a potential risk. Contamination with synthetically derived templates, such as amplicons, is an inherent weakness of PCR and can be mitigated by physically separating specimen preparation from analysis [12].

Other synthetically derived contamination can occur if demand increases for synthesised oligonucleotides as positive controls [3], which is a popular initial route used to support assay development for new pathogens in non-endemic regions (see below). For monkeypox, contamination should be monitored by individual laboratories with whole-process negative pathogen controls that ideally contain human genomic DNA. As a variable technical artefact that will differ between laboratories, contamination should be treated outside normal sensitivity or specificity assessment.

## Routes to ensure test accuracy

The clinical accuracy of monkeypox PCR assays will be determined by the clinical community and influenced by how they are used in terms of disease stage and specimen choice. Specimens typically comprise swabs of skin lesions and oropharynx but may extend to a range of other specimens for research purposes [13]. Pre-analytical factors including specimen and swab choice, method of obtaining specimens, transport media and extraction method can vary in performance, influencing the quantity and quality of nucleic acid available to the final PCR step and therefore also the diagnostic performance. Consequently, these steps should be considered when evaluating characteristics such as the LOD. 

Positive monkeypox virus controls are important in providing test confidence, but can also be used to determine analytical performance, such as LOD. However, they come in a range of different formats that vary in suitability for such assessments. In the short term, synthetic molecules (mentioned above) provide quick-access positive controls for the PCR step, but they cannot control for other parts of the diagnostic process, such as the extraction step used to purify nucleic acids, and are therefore of limited value when exploring factors like the LOD of a diagnostic pipeline. With time, whole-viral control materials, or virus-like materials such as pseudo-viruses, typically become established to provide a more comprehensive route for harmonising clinical diagnostic pipelines. Such materials are therefore preferred for a more accurate assessment of analytical performance characteristics like LOD, once widely available. 

While cultured virus is preferable to purified nucleic acid, it is less suitable for controlling for differences in specimen type, heterogeneity or sampling. As noted during the COVID-19 pandemic, routes to better standardise diagnostic protocols that use disparate and heterogenous specimens remain an important yet challenging area of research and development. Work on this topic will hopefully improve diagnostic test accuracy for diseases that are more far-reaching than monkeypox or COVID-19. 

As PCR detection of SARS-CoV-2 RNA became more established and as the pandemic evolved, the importance of sequencing to identify and track variants of concern became clear. This was epidemiologically important but also predicted the impact of genetic changes on assay performance and potential false-negative results. While monkeypox virus may not evolve as quickly as SARS-CoV-2, it should not be assumed to be slow evolving [14] and its genome should be monitored during the epidemic. There are already genetic changes to some of the sequences targeted by published PCRs in the monkeypox virus that is currently in circulation; they are highlighted in the Table and additional detail is provided in the Supplement.

While quantitation of monkeypox virus DNA may not be used to direct clinical decisions, quantitative considerations are required to evaluate analytical performance, such as LOD, and facilitate demonstration of competencies to meet regulatory requirements and in response to industry target product profiles. Furthermore, monkeypox virus genome quantities, and how they change during the course of infection, will probably be used to attempt to understand disease dynamics and infer disease stage or infectiousness. Such assessments of virus quantities and their epidemiological or clinical implications must rely on adequate methodology, taking into consideration the impact of pre-analytical factors outlined above and careful standardisation [15]. 

Quantitative PCR (qPCR) is automated and consequently a popular diagnostic format. It provides a crude quantitative output in the quantification cycle (C_q_) (also termed cycle threshold (C_t_)), but uncalibrated C_q_ should not be used as a unit to estimate quantities, set thresholds or report LOD [15]. Copy-based units of viral genome (per appropriate unit volume, e.g. mL) are preferred over C_q_ for quantification. However, users need to select appropriate standard materials for determining copies as available positive control materials may not have been accurately certified for quantitation if they are not intended for use as calibrators. Reference materials that have been comprehensively characterised in terms of quantity, stability, batch variability etc. are key for ensuring accurate traceable evaluation of test performance in support of harmonisation and determination of characteristics such as LOD. 

Early in an epidemic, diagnostic standards may be scarce, and methods such as digital PCR (dPCR) can act as a reference as they can provide high quantitative accuracy without calibration [16]. In its current format, dPCR may not be the first choice as a diagnostic solution when responding to an epidemic because other formats of PCR can offer higher throughput and are more widely available and established in clinical laboratories. However, dPCR was used to support routine diagnostic PCR during COVID-19 by providing an anchor value to which diagnostic formats could be compared [17]. Using published PCR assays, dPCR can be applied to monkeypox virus DNA measurements with high reproducibility across platforms as illustrated in the Figure comparing uncalibrated measurement of two different assays using two instruments (experimental details are available in the Supplement). It could be used to determine (or ‘value assign’) the quantity of monkeypox virus control materials (in genome copies or equivalent) used by routine laboratories to ensure their diagnostic PCRs perform within specification.

**Figure:**
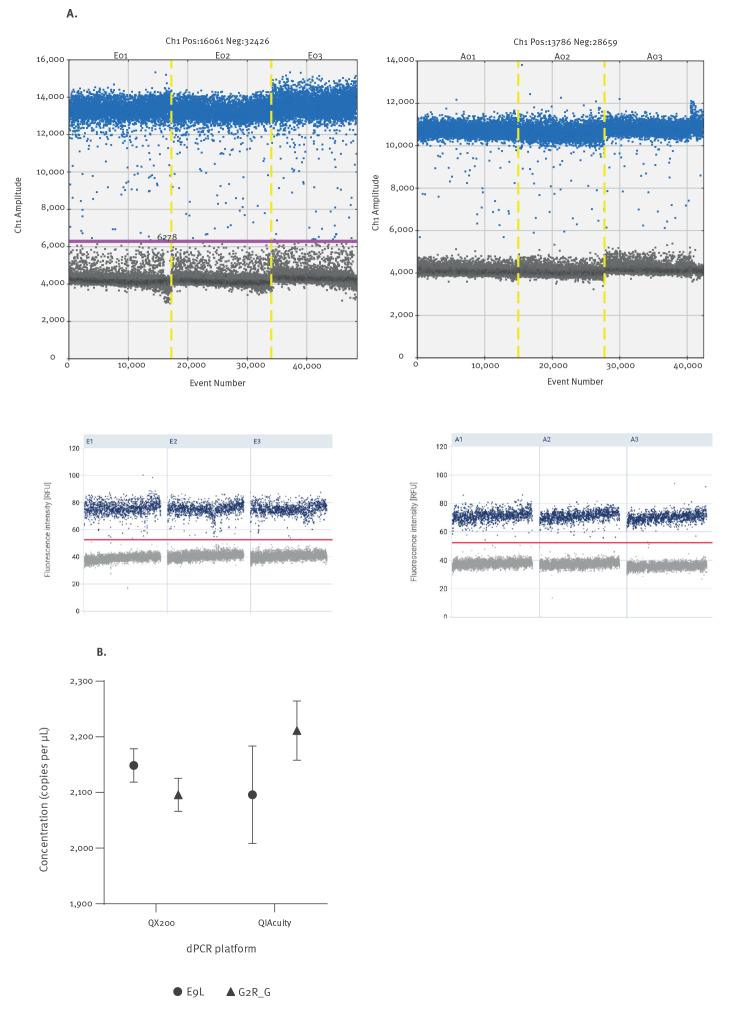
Example of monkeypox dPCR assay conducted without calibration

High reproducibility demonstrated when varying primer sets (E9L [10] and G2R_G [9]) and instrument (QX200 (Bio-Rad) or QIAcuity One (Qiagen)) using a synthetic DNA template containing both gene sequences (ca 1,250 copies/µL estimated using Qubit Fluorometer 2.0 (ThermoFisher Scientific) with Qubit dsDNA BR assay kit).

Panel A: One-colour plots of the respective reactions and instruments. Positive partitions, containing amplified template, are coloured in blue and negative partitions, indicating no template are coloured in grey.

Panel B: Quantitative outputs of the respective assays and instruments (mean ± 1 SD). Ordering and establishing the assay took less than 3 weeks, illustrating how this approach can be rapidly deployed to provide quantification of standard materials in support of routine diagnostics or clinical samples to provide accurate reference ranges of viral quantity. The corresponding dMIQE table and reaction output data for each instrument can be found in Supplementary Tables S2 and S3, respectively.

## Guidance to aid monkeypox PCR testing

The WHO interim guidance (published in May 2022) is very timely for the efforts of harmonising monkeypox diagnostics [13]. The guidance stipulates that the diagnostic PCRs for monkeypox should include positive controls at low (above the LOD) but easily detectable concentration. As written, users from two different laboratories could follow this guidance but still differ considerably in their experimental design (e.g. one considering just the PCR step for LOD and the other the wider experimental process) or suitability of control materials used to determine analytical performance. This could lead, to some extent, to test discrepancies which in turn vary in diagnostic performance despite both laboratories ‘following’ the guidance. What is ‘easily detectable’ in terms of a monkeypox infection is yet to be determined and there is also a need to decide when that criterion has been met and that will depend on accurate PCR methodologies to deliver robust measurements to guide those decisions. 

The WHO guidance further suggests that controls should provide information about sample and nucleic acid quality as well as an evaluation of the success of the diagnostic pipeline (to ensure that processes such as nucleic acid extraction have worked properly). While the latter may be readily addressed by including internal process controls, metrics for specimen and nucleic acid ‘quality’ are not easy to deliver. The monkeypox protocol from the United States Centers for Disease Control and Prevention (CDC), published in June 2022 [18], is adapted from an earlier publication [10] and incorporates a human genetic target (RNase P) as an internal positive control to monitor false negative results. 

A human target can control for specimen and nucleic acid quality as well as diagnostic pipeline, yet how well this can control diagnostic performance is often undefined and will vary with specimen type and quality. Alternative internal positive controls, such as spiked cultured viruses, control for processes like lysis and nucleic extraction, but not specimen quality, are preferable to pure nucleic acid spike controls. Nucleic acid internal controls, while popular, cannot control for preanalytical steps such as viral lysis. The appropriateness of different internal controls can be evaluated experimentally by comparing their performance with positive clinical specimens or contrived samples. This can be achieved by simulating poor extraction, inclusion of inhibitors, etc. although based on current literature such assessment of internal control suitability is seldom performed. 

The importance of the considerations specified by the WHO in terms of clinical relevance may vary for monkeypox diagnosis with different specimen types. The quantity of virus in clinical specimens will impact on how easily a test protocol can detect viral genomes, with high amounts simplifying detection but concomitantly increasing the risk of false-positive results because of cross contamination. Reports on quantities of monkeypox virus in specimens are limited. In a study of seven patients by Alders et al. 2022 the PCR C_q_ rarely dropped below cycle 20 [19]. As C_q_ can vary 1,000-fold [15], it is difficult to know whether this cycle 20 represents a large concentration of virus (≥ 10^9^ genome copies/mL) easily detected even with lower-sensitivity test formats, a lower titre (≤ 10^6^ genome copies/mL) making increased analytical sensitivity more important, or somewhere in between. Future studies exploring the quantities of monkeypox virus DNA must provide an estimation of how a given C_q_ value corresponds to viral genome copies if this work is to be used to aid the diagnostic discussion. Moreover, the correlation between virus quantity in various specimens and infectiousness needs to be established if PCR is to be used for risk assessment and not only virus detection. The C_q_ value alone provides limited information on which to make such assessments.

Wider guidance on how to approach and deploy diagnostic PCRs has also been provided in a number of documentary standards and technical specifications from the International Standards Organisation (ISO) technical committees 212 (Clinical laboratory testing and in vitro diagnostic test systems) and 276 (Biotechnology) which directly address molecular measurement. Examples include technical specifications on SARS-CoV-2 [20] and more broader diagnosis of microbial pathogens [12]. The COVID-19 pandemic has also driven the measurement science and diagnostic community to work together to explore how test accuracy can be best ensured when responding to a pathogen of epidemic potential [21]. Many of the recommendations in response to COVID-19 are directly applicable to monkeypox.

## Conclusion

A notable legacy of the COVID-19 pandemic is the application of diagnostic PCR at an unprecedented global scale and an enhancement of molecular diagnostic capacity. The infrastructure, expertise and important experience gained during the COVID-19 response are likely to result in rapid development and adoption of molecular assays for other emerging or re-emerging threats. Should the monkeypox epidemic require testing to be deployed even more widely and sustainably, a focused effort to standardise the laboratory response and implement the lessons learned from COVID-19 should be prioritised. Such a proactive response will not hinder any diagnostic response; the opposite is more likely. COVID-19 has taught us that it is not acceptable to assume detection is simple because the viral load (and thus genome copies) can be high. A cautious approach to deploying PCR tests for monkeypox is likely to improve their impact and ensure the trust of the scientific community in this approach is sustained. This will empower those tasked with responding to the epidemic with the most accurate information to guide decisions across the whole spectrum from patient management to public health policy.
